# Supplementary material for: Purification of micrococcal nuclease for use in ribosomal profiling of high-salinity extremophiles
Source: J Biol Chem. 2024 Nov 27;301(1):108020. doi: 10.1016/j.jbc.2024.108020 (PMC11719836; doi:10.1016/j.jbc.2024.108020)
Supplement: Supporting Information [file mmc1.pdf]

Supporting Material

Purification of micrococcal nuclease for use in ribosomal profiling of high-salinity extremophiles

Pavlina Gregorova[1], Matthew Isada[2], Jocelyne DiRuggiero[2], L. Peter Sarin[1,*]

[1] RNAcious Laboratory, Molecular and Integrative Biosciences Research Programme, Faculty of Biological and Environmental Sciences, University of Helsinki, Viikinkaari 9B, 00790 Helsinki, Finland

[2] Department of Biology, Johns Hopkins University, 3400 N. Charles St., 235 Levi Hall, Baltimore, MD 21218 Baltimore, Maryland, USA

[*]To whom correspondence should be addressed: peter.sarin@helsinki.fi, +358-2941-59533

Running title: Production of MNase for ribosomal profiling

KEYWORDS

Micrococcal nuclease, S7, MNase, ribosome profiling, haloarchaea, halophiles, archaea

Supporting Materials and Methods

Site-directed mutagenesis of MNaseB to prepare MNaseA construct

Briefly, the PCR reaction contained 0.5 µM of each primer, 0.4 mM dNTPs, 1x HF buffer, 0.02 U/µL Phusion polymerase (ThermoFisher Scientific, # F-530L) and 2 ng template. The mutagenesis was carried out using touchdown PCR: initial denaturation at 98 °C for 5 min, 20 cycles of 98 °C for 20 s, 60-50 °C (step -0.5°C/cycle) for 20 s, 72 °C for 1 min 30 s; 10 cycles of 98 °C for 20 s, 55 °C for 20 s, 72 °C for 1 min 30 s; final extension at 72 °C for 10 min and stored at 8 °C. Following the PCR, 2 µL of the unpurified PCR product was circularized by performing kinase/ligase mixture [0.5x T4 DNA ligase buffer, 0.5x T4 PNK buffer, 0.5 mM ATP, 0.5 U/µL T4 DNA Ligase (ThermoFisher Scientific, #EL0014), 1 U/µL DpnI (ThermoFisher Scientific, #FD1703), 0.5 U/µL T4 PNK (ThermoFisher Scientific, #EK0031)]. Reaction was incubated at 22 °C for 2 h, followed by inactivation at 70 °C for 5 min and transformed to chemically competent *E. coli* DH5α. The colonies were screened by colony PCR and propagated in *E. coli* DH5α in LB supplemented with 50 µg/mL kanamycin. Sequence was verified by Sanger sequencing (Eurofins).

Cell lysis using sonication

As an alternative to cell lysis by cryomill, the sonication was equally efficient lysis method. For sonication, harvested cells were snap frozen as wet pellets, weighted, and stored at -80 °C until further processed. Then, the pellets were melted on ice and 5 mL of Lysis buffer was added per 1 g of wet pellet and cells were resuspended by pipetting. Sonication (Sonicator UP400 S Dr. Hielscher) was done using the following program: 10 cycles, 60% amplitude, 0.5 cycle, 20 s ON/1 min OFF. Lysate was centrifuged for 10,000 x *g,* 30 min, 4 °C. The resulting supernatant was used for purification as described for MNaseA.

MNase B purification

The purification is performed in same manner as for MNase A. However, the purification buffers have slightly different composition. The composition of these buffers is below:

- MNase B lysis buffer (**LB-MNB**) [50 mM Tris-HCl (pH 7.5), 500 mM NaCl, 1 mM $CaCl_2$, 5 mM imidazole (pH 7.5), 5% (vol/vol) glycerol, 1× Halt protease inhibitor (ThermoFisher Scientific #87786)]
- MNase B wash buffer (**WB-MNB**) [50 mM Tris-HCl (pH 7.5), 500 mM NaCl, 10 mM imidazole (pH 7.5) and 5% (vol/vol) glycerol]
- MNase B elution buffer (**EB-MNB**) [50 mM Tris-HCl (pH 7.5), 500 mM NaCl, 250 mM imidazole (pH 7.5) and 5% (vol/vol) glycerol]

MNase A expression in *E. coli* Lemo21(DE3)

To increase the OmpA, MNaseA can be also expressed in *E. coli* Lemo21(DE3) using pET28a(+)-OmpA-MnaseA plasmid. For expression, freshly transformed *E. coli* Lemo21(DE3) cells were inoculated to LB-Luria (10 g/L tryptone, 5 g/L yeast extract, 0.5 g/L NaCl supplemented with 50 µg/mL kanamycin, 35 µg/mL chloramphenicol) and grown overnight at 37 °C with shaking at 200 rpm. The stationary cultures were diluted to OD_{600} = 0.1, supplemented with antibiotics and 500 µM L-rhamnose and grown to an OD_{600} of 0.4-0.5, at which point protein expression was induced by IPTG (ThermoFisher Scientific, #R0392) addition (1 mM final concentration) and subsequently carried out at 30 °C with shaking at 200 rpm for 9-10 h. The cells were collected by centrifugation at 5,000 x *g* for 15 min at 4 °C and processed as described in Methods section.

Supporting Figures

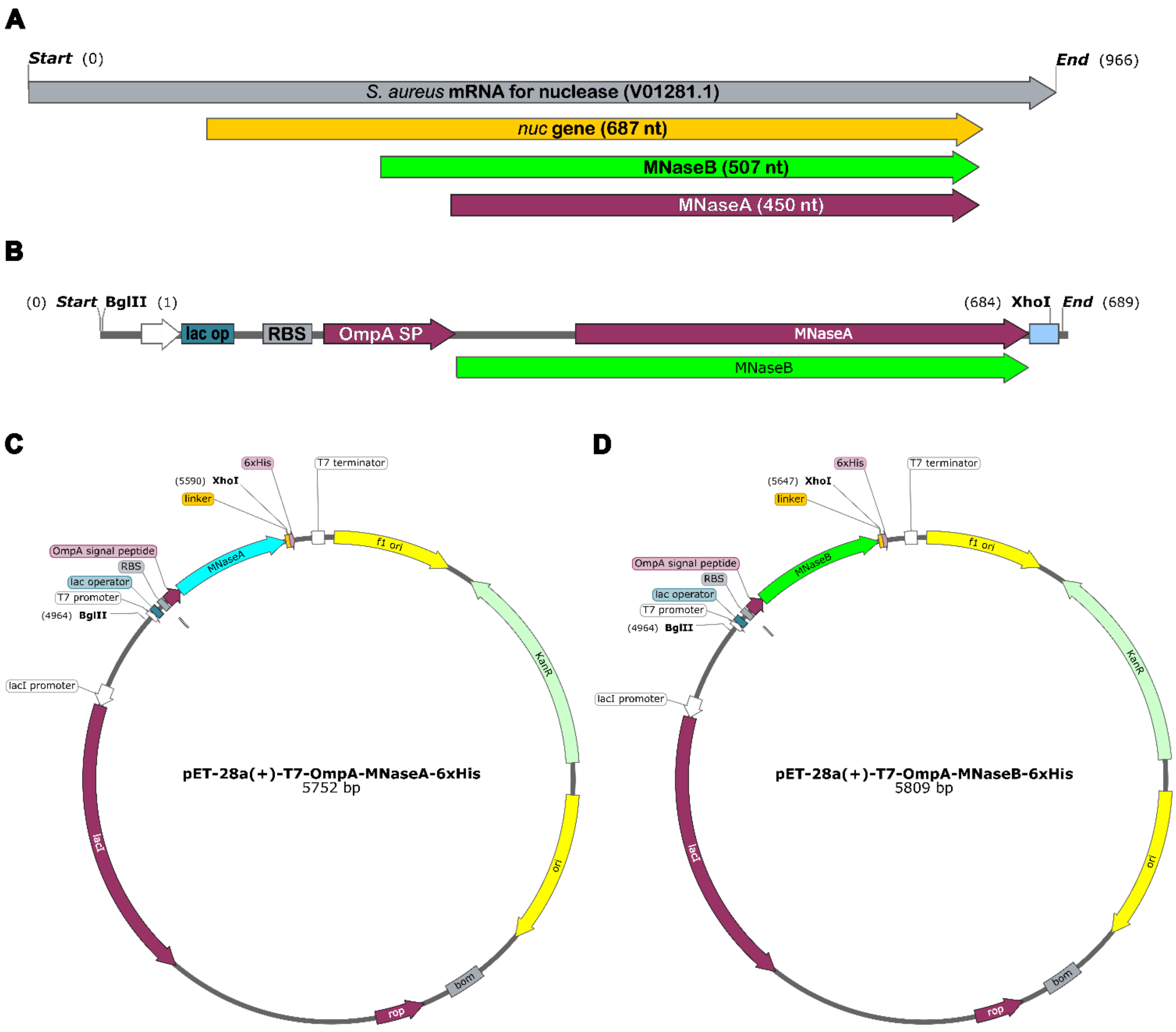

Supporting Figure S1. (A) Schematic view of *S. aureus* mRNA for nuclease (966 nt) organization (GenBank no. V01281.1). *Nuc* gene is encoded by 687 nt (226-912 nt) of which MNaseB is 507 nt (403-912 nt) and MNaseA is 450 nt (463-912 nt). **(B)** Schematic view of synthetic gene construct for MNase production. Plasmids designed in the study. **(C)** Plasmid for expression of MNaseA and **(D)** MNaseB, respectively.

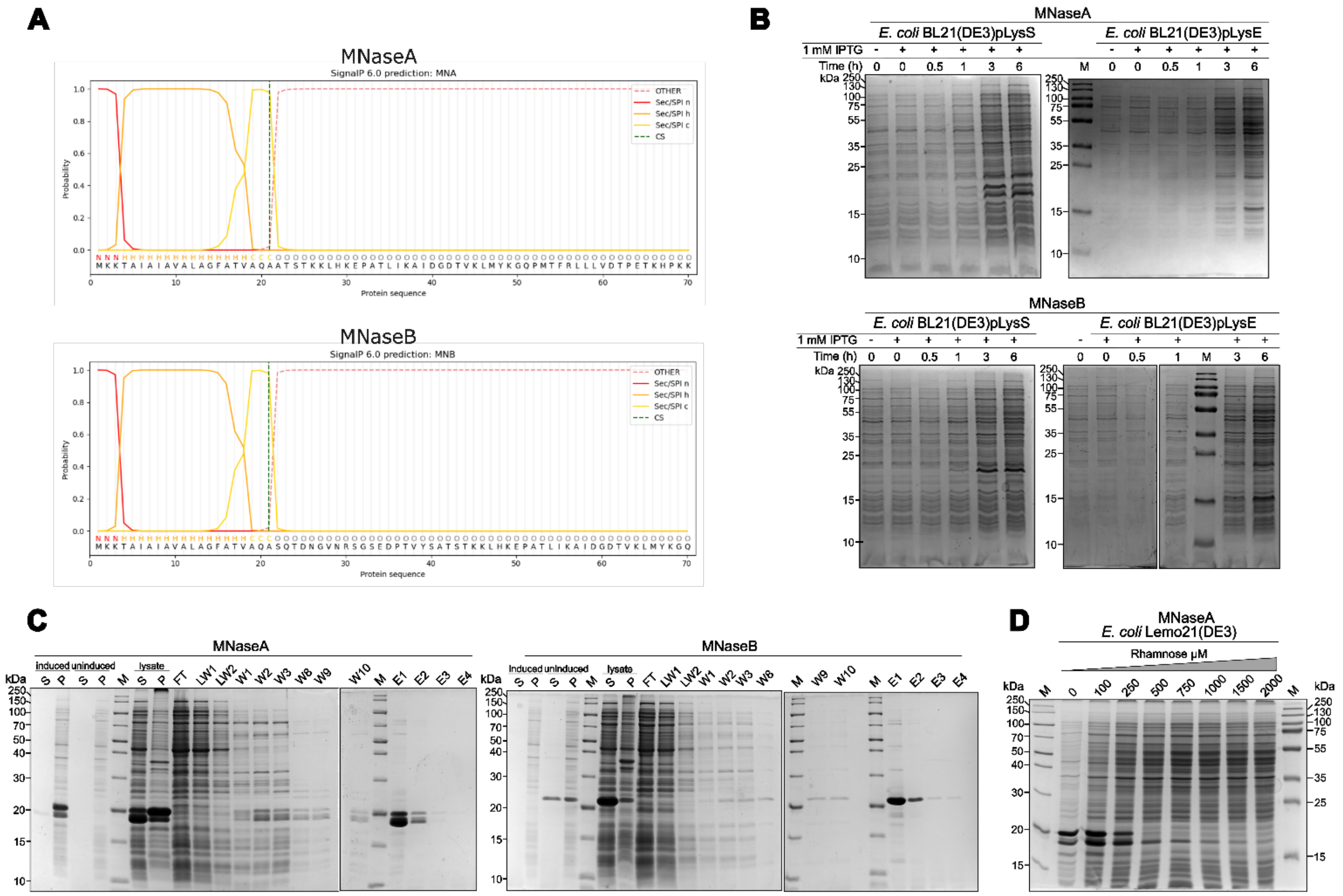

Supporting Figure S2. (A) Prediction of cleavage probabilities of OmpA signal peptide from MNaseA and MNaseB. Values calculated by SignalP ver 6.0. **(B)** Expression of MNaseA (top) and B (bottom) at different time points in two different *E. coli* expression strains. M – protein marker. **(C)** SDS-PAGE analysis of fractions from purification of MNaseA (left) and B (right). Induced – cultures with 1 mM IPTG, uninduced – no IPTG, S – supernatant, P – pellet, FT – flowthrough, LW – lysis buffer wash, W – wash, E – elution. **(D)** Optimization of MNaseA expression in *E. coli* Lemo21(DE3) strain by increasing rhamnose.

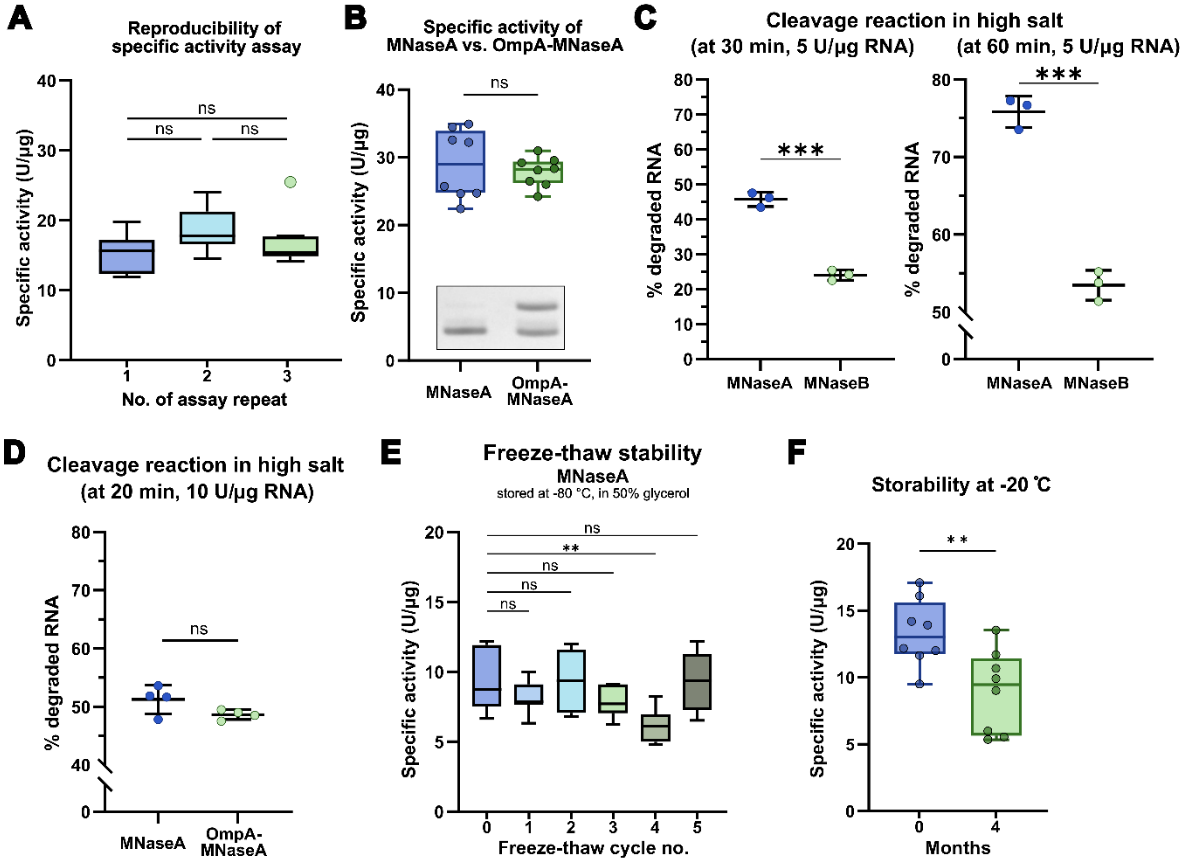

Supporting Figure S3. (A) Comparison of specific activity assays from three independent replicates done with commercial MNase. Ordinary one-way ANOVA (N=8, ns – not significant). **(B)** Specific activity of purified MNaseA and OmpA-MNaseA. The insert shows the purity of each tested MNaseA. Unpaired two-tailed Student's t-test (technical replicates, N=8, ns – not significant) **(C)** Densitometrical quantification of MNaseA and B activity in high salt reactions using 5U of enzyme per 1 µg of RNA at 30 min (left panel) and 60 min (right panel). Samples were compared by unpaired two-tailed t-test (technical replicates, N=3; *** p<0.001). **(D)** Densitometrical quantification of MNaseA and OmpA-MNaseA (same proteins as in panel B) activities in high salt reactions using 10U of enzyme per 1 µg of RNA after 20 min reaction. Samples were compared by unpaired two-tailed Student's t-test (technical replicates, N = 3; *** p<0.001). **(E)** Freeze-thaw stability of the purified MNaseA stored at -80 °C in storage buffer containing 50% glycerol. Samples were compared by ordinary one-way ANOVA (N=8, ** p=0.0039). **(F)** Stability of the purified MNaseA at -20 °C for 4 months in storage buffer containing 50% glycerol. Unpaired two-tailed Student's t-test (technical replicates, N=8, ** p=0.0075). Error bars in all panels represent standard deviation.

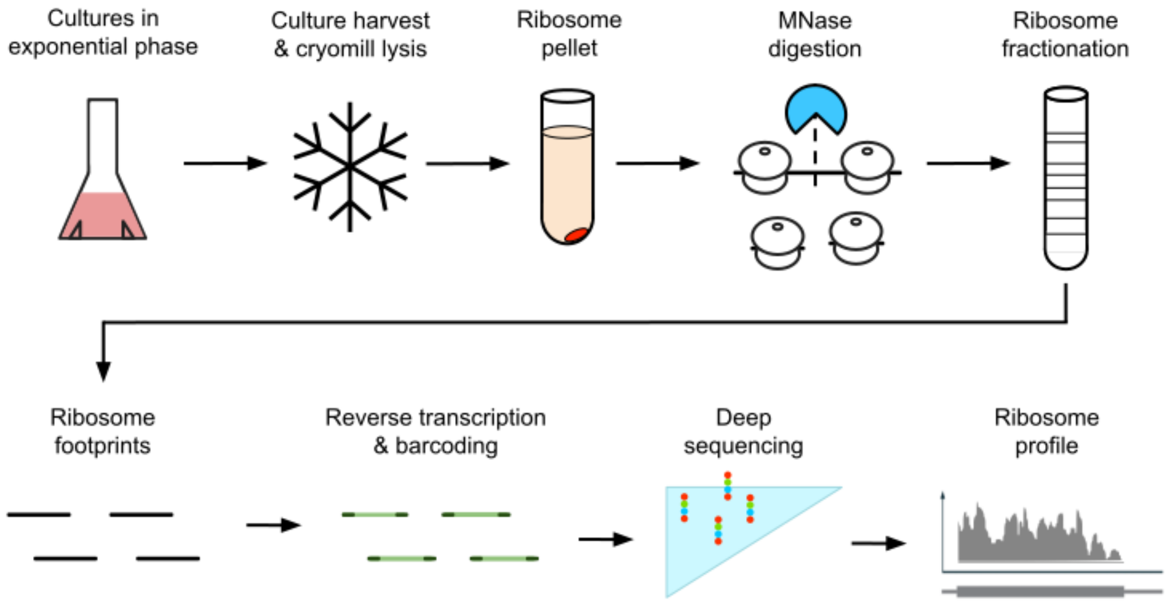

Supporting Figure S4. Workflow schematic for ribosome profiling in *Haloferax volcanii*. Cells were harvested in exponential phase by flash-freezing cultures dropwise and cryogenically milled (cryomilling) in liquid nitrogen. Ribosomes were pelleted from thawed lysate by ultracentrifugation, treated with MNase to digest unprotected mRNA, and separated along a 10-50% sucrose gradient; during these steps, ribosome pellets were processed in lysis buffer containing 3.4 M KCl to keep 70S monosomes intact. Ribosome footprints were then isolated from 70S sucrose fractions, reverse transcribed, barcoded with sample-specific index sequences, and sequenced.

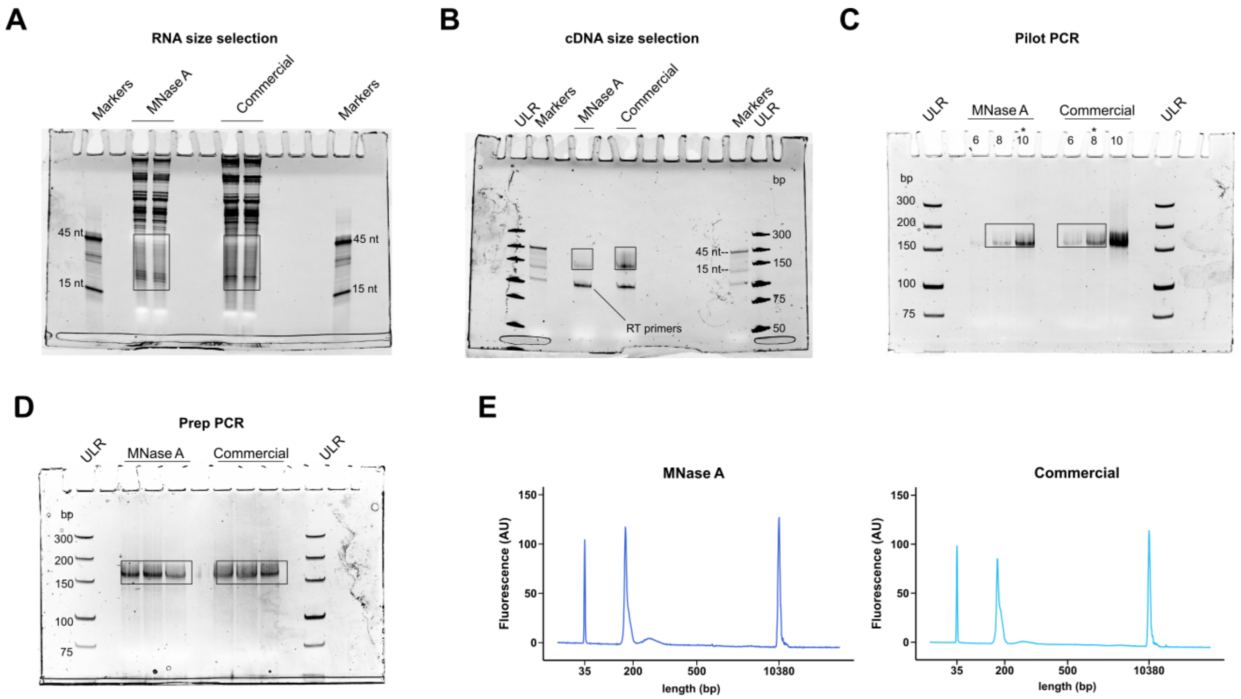

Supporting Figure S5. Ribosome profiling library construction steps and quality control for samples digested with MNase A or commercial MNase. Polyacrylamide gels used for **(A)** RNA footprint size selection, **(B)** barcoded/reverse-transcribed footprint size selection, **(C)** pilot PCR (cycle number selected for preparative PCR marked by (*)), and **(D)** preparative PCR. **(E)** Bioanalyzer traces of completed ribosome profiling libraries; a peak at the expected size for each final library is at ~170 bp. ULR: Ultra Low Range DNA ladder. One out of three replicates for each enzyme are shown here.

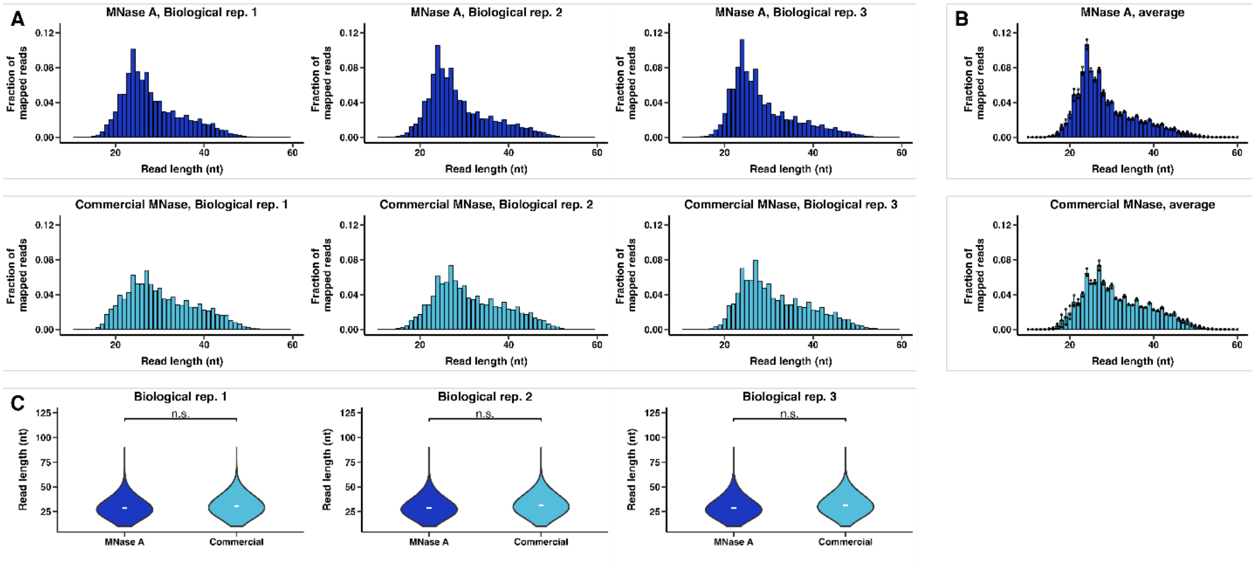

Supporting Figure S6. Read length distributions of ribosome profiling libraries digested with MNase A (dark blue) or commercial MNase (light blue). **(A)** Read length distributions for each of three replicates; reads per length were normalized to the total number of genome-mapped reads. **(B)** Read length distributions from MNase A and commercial MNase averaged across biological three biological replicates each; error bars indicate standard deviation. Individual biological replicates represented by points. **(C)** Read length distributions for each of the three replicates as violin plots; distribution means are marked by (-). Statistical comparison between distributions in each biological replicate (permutation test with distribution mean as test statistic, 10,000 resamples, independent) showed no significant difference (p-values: biological rep. 1, p-value = 0.7677; biological rep. 2, p-value = 0.7249, biological rep. 3, p-value = 0.6657).

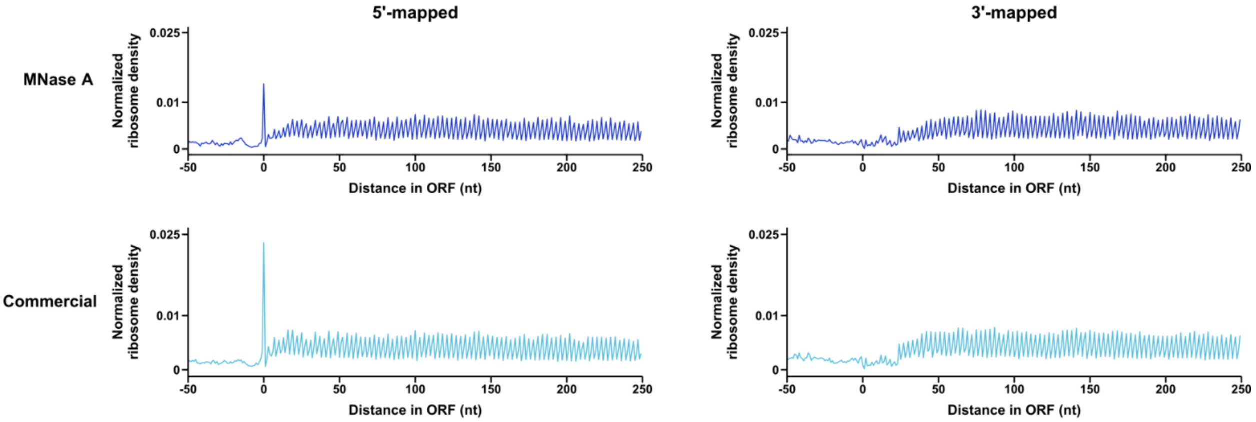

Supporting Figure S76. Metagene plots of ribosome profiling libraries for a single biological replicate digested with MNase A (top, dark blue) or commercial MNase (bottom, light blue). Metagene plots show the mean ribosome density on all genes aligned at their start codon using 5'-end or 3'-end mapped reads. Note that left-side panels (5'-mapped reads) are a reproduction of Figure 3D and are included here to facilitate comparison to 3'-mapped reads.

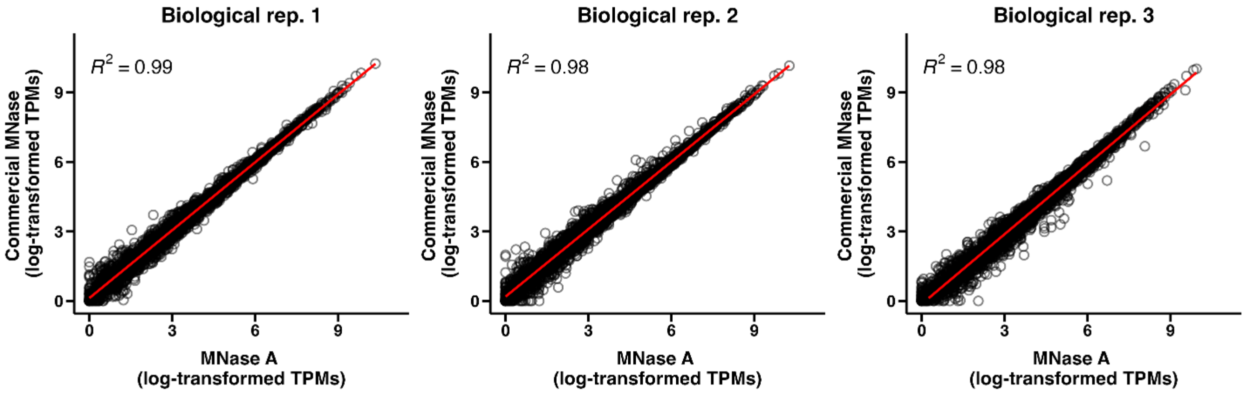

Supporting Figure S8. Correlation plots of ribosome occupancy per gene between samples treated with MNase A (x-axis) and commercial MNase (y-axis) within each of three biological replicates. For each gene, ribosome occupancy data from ribosome profiling were converted to transcripts per million (TPMs), log-transformed, and compared between MNase treatments.
